# Dynamic changes in proresolving lipid mediators and their receptors following acute vascular injury in male rats

**DOI:** 10.14814/phy2.16178

**Published:** 2024-08-11

**Authors:** Hideo Kagaya, Alexander S. Kim, Mian Chen, Pei‐Yu Lin, Xuanzhi Yin, Matthew Spite, Michael S. Conte

**Affiliations:** ^1^ Cardiovascular Research Institute and Department of Surgery University of California San Francisco San Francisco California USA; ^2^ Center for Experimental Therapeutics and Reperfusion Injury, Department of Anesthesiology, Perioperative and Pain Medicine Brigham and Women's Hospital and Harvard Medical School Boston Massachusetts USA

**Keywords:** angioplasty, inflammation, lipid mediators, neointima, resolution

## Abstract

Acute vascular injury provokes an inflammatory response, resulting in neointimal hyperplasia (NIH) and downstream pathologies. The resolution of inflammation is an active process in which specialized proresolving lipid mediators (SPM) and their receptors play a central role. We sought to examine the acute phase response of SPM and their receptors in both circulating blood and the arterial wall in a rat angioplasty model. We found that the ratio of proresolving to pro‐inflammatory lipid mediators (LM) in plasma decreased sharply 1 day after vascular injury, then increased slightly by day 7, while that in arteries remained depressed. Granulocyte expression of SPM receptors ALX/FPR2 and DRV2/GPR18, and a leukotriene B4 receptor BLT1 increased postinjury, while ERV1/ChemR23 expression was reduced early and then recovered by day 7. Importantly, we show unique arterial expression patterns of SPM receptors in the acute setting, with generally low levels through day 7 that contrasted sharply with that of the pro‐inflammatory CCR2 receptor. Overall, these data document acute, time‐dependent changes of LM biosynthesis and SPM receptor expression in plasma, leukocytes, and artery walls following acute vascular injury. A biochemical imbalance between inflammation and resolution LM pathways appears persistent 7 days after angioplasty in this model. These findings may help guide therapeutic approaches to accelerate vascular healing and improve the outcomes of vascular interventions for patients with advanced atherosclerosis.

**NEW & NOTEWORTHY**

This study demonstrates the acute phase response of lipid mediators including the specialized proresolving mediator (SPM) and their receptors in both circulating blood and within the arterial wall following vascular injury. The findings have implications for novel approaches to promote healing and reduce neointimal disease following vascular interventions.

## 1 INTRODUCTION

Acute vascular injury induces a classic wounding response including an immediate thrombo‐inflammatory phase, followed by resolution and tissue remodeling (Conte et al., 2018; Wu, Mottola, Schaller, et al., 2017). The final remodeling stage generally results in the formation of neointimal hyperplasia (NIH), which may lead to vessel narrowing (de Vries et al., 2016; Satish & Agrawal, 2019). In the clinical context, common interventions for atherosclerosis such as balloon angioplasty, stenting, and bypass surgery are relevant examples whose long‐term efficacy remains significantly limited by NIH (Conte et al., 2006). The spatial and temporal extent of this response varies by the nature of the injury (e.g., acute mechanical stretch versus implant), the underlying vascular pathology, and the species studied. Current approaches to deal with this highly prevalent clinical problem focus on anti‐proliferative and anti‐inflammatory agents, with notable limitations. Given that atherosclerosis remains a dominant cause of mortality and morbidity around the globe, new approaches to reduce NIH and extend the benefits of these life‐ and limb‐saving interventions are critical.

The resolution of inflammation is now understood as an active biologic process with specific mediators, receptors, and cell‐signaling events (Serhan & Savill, 2005). Studies in small animal models of sterile inflammation led to the elucidation of new classes of bioactive lipid mediators (LM) derived from polyunsaturated fatty acids (PUFA) collectively termed as specialized proresolving mediators or SPM (Serhan, 2014). This represents a defined LM class switch from the hyperacute phase of arachidonic acid‐derived eicosanoids (prostaglandins and thromboxanes) to alternative products largely derived from n‐3 PUFA (e.g., docosahexaenoic and eicosapentaenoic acids) (Levy et al., 2001; Serhan et al., 2004). SPM regulate leukocyte phenotype and interactions, attenuating pro‐inflammatory signals, and promoting local clearance of debris, efferocytosis, and tissue remodeling. Several families of SPM have now been characterized including the lipoxins, D‐ and E‐resolvins, protectins, and maresins (Serhan, 2014; Serhan & Petasis, 2011). Stereochemical assignments and total organic syntheses have been provided for an increasing number of SPM (Chiang & Serhan, 2020; Dalli, Colas, & Serhan, 2013; Schwab et al., 2007; Serhan, 2017; Serhan et al., 2015). SPM signaling occurs largely through interactions with cell‐surface receptors. Multiple specific SPM receptors have now been identified, belonging to the G‐protein coupled receptor (GPCR) superfamily (Chiang & Serhan, 2017).

There is a growing appreciation for the role of SPMs in vascular disease (e.g., atherosclerosis, aneurysm, thromboembolism), injury, and repair (Fredman & Spite, 2017; Kim & Conte, 2020; Pirault & Back, 2018). Prior work has shown that SPM may be detected in the circulation of patients with vascular disease, following surgical interventions, and may be altered by nutritional supplementation with marine oils (Schaller et al., 2020). Human arterial tissue can synthesize bioactive SPM when precursor free fatty acids are provided (Chatterjee et al., 2017). Studies in animal models of arterial injury or vascular grafting (mouse, rat, rabbit) have shown that SPM treatment attenuates NIH formation although precise mechanisms, and pharmacokinetic requirements, remain unclear (Akagi et al., 2015; Kim et al., 2022; Miyahara et al., 2013; Wu et al., 2018; Wu, Mottola, Chatterjee, et al., 2017). In this regard, little is known about the expression and function of the SPM receptors in the context of vascular injury. We undertook the present study to describe the acute phase LM response in circulating blood and the arterial wall using an established rat angioplasty model, and to characterize both SPM and SPM receptor expression within these distinct compartments. Our results demonstrate early, dynamic changes in LM circuits that may drive the critical conversion from inflammation to resolution in arterial injury.

## 2 MATERIALS AND METHODS

### 2.1 Animal model: Rat carotid artery angioplasty

We used Male Spraque‐Dawley rats (300–400 g) in compliance with an Institutional Animal Care and Use Committee approved protocol (University of California, San Francisco). We produced arterial injury by balloon angioplasty via midline cervical incision to the left common carotid artery, as previously described (Kim et al., 2022; Wu, Mottola, Chatterjee, et al., 2017). Specifically, we cannulated the left common carotid artery, inserted a 2F balloon (Edwards Lifesciences, Irvine, Calif) through the external carotid artery and inflated the balloon to 5 atm for 1 min using a calibrated balloon inflation device (Boston Scientific, Marlborough, Mass). After angioplasty, we ligated the external carotid artery and restored flow. We used the contralateral carotid artery for sham surgery, in which we exposed and encircled the artery but did not insert the balloon into it. We used the intact carotid arteries, thoracic aortas, hearts, lungs, spleens, and cervical lymph nodes as uninjured controls. Animals were euthanized with isoflurane, carbon dioxide, and bilateral thoracotomies at 4 and 24 h, 3 and 7 days after angioplasty. Blood samples were collected from tail veins at each time point after angioplasty. We harvested tissues after perfusion with heparinized saline for mRNA analysis. For immunofluorescence analysis, tissues were fixed with 4% formaldehyde.

### 2.2 Monocyte adhesion assay

We utilized HUVECs, grown to 100% confluency on coverslips in 24 well plate, for an in‐vitro monocyte adhesion model as previously described (Patricia et al., 1999). Briefly, HUVECs were activated with TNFα at indicated doses for 4 h. U937 monocytes were labeled with Cell Trace CFSE (Thermo Fisher Scientific, Waltham, MA) for 30 min at 37°C. We added labeled monocytes to HUVECs after 4 h of TNFα incubation and allowed them to incubate in serum free media at 37°C for 4 h. HUVECs were washed three times with PBS and treated with lysis buffer for RNA analysis or fixed in 4% paraformaldehyde (PFA) for immunocytochemistry analysis.

### 2.3 Targeted liquid chromatography tandem mass spectrometry (LC–MS/MS) analysis of lipid mediators

Plasma and carotid arteries were harvested at baseline, 1 and 7 days after angioplasty or sham surgery for targeted LC–MS/MS analysis of LM. Tissues were immediately frozen after harvest. Methanol containing deuterated internal standards (d4‐LTB_4_, d8‐5‐HETE, d4‐PGE_2_, d5‐LXA_4_, d5‐RvD2, d5‐MaR1; Cayman Chemical, Ann Arbor, MI) was added to the samples. Tissue was minced with surgical scissors on ice and samples were placed at −80°C to allow for protein precipitation. Supernatants were collected after centrifugation at 13,000 rpm for 10 min at 4°C. Subsequently, LM were extracted using solid‐phase C18 columns, as previously described (Dalli et al., 2018). Methyl formate fractions were collected, the solvent was gently evaporated using N_2_ gas, and samples were resuspended in methanol: water (50:50, v/v). LC–MS/MS analysis was carried out using a Shimadzu HPLC system that was equipped with a reverse‐phase C18 column (Poroshell 100 mm × 4.6 mm × 2.7 mm; Agilent Technologies) that was held at 50°C and coupled to a Qtrap 5500 mass spectrometer (AB Sciex, Framingham, MA). A gradient of methanol–water‐acetic acid ranging from 50:50:0.01 (v/v/v) to 98:2:0.01 (v/v/v) was used at a flow rate of 0.5 mL/min. The mass spectrometer was operated in negative ionization mode and LM were identified using multiple reaction monitoring (MRM) transitions that were optimized for each lipid mediator using synthetic standards. Identification and quantification of LM in samples was accomplished by matching retention time and MS/MS spectra with synthetic standards run in parallel and by interpolating MRM peak areas above the baseline to external standard curves for each mediator. Extraction recovery was accounted for using internal deuterated standards with similar retention times. The lower limits of quantification were calculated using standards of each mediator class by determining the amount that could be quantified in replicate injections with a coefficient of variation of less than 20%. Levels of LM were normalized to plasma volume or protein content of the tissue taken for extraction.

### 2.4 SPM receptor expression on circulating leukocytes

Whole blood samples were obtained in heparin. Blood was divided into aliquots and treated with Fc Block, Live/Dead (Zombie Aqua™ Fixable Viability Kit 423,102, Biolegend) and the following leukocytes markers (2.5 μL per sample): CD4‐Super Bright 436 (62–0040‐82, eBioscience), CD3PE (12–0030‐82, eBioscience), CD11b/c‐PE/CY7 (201,818, Biolegend), CD45RA‐APC (47–0462‐82, eBioscience), CDCD161‐PerCP‐Efluor 710 (46–1610‐80, eBioscience) and receptor markers or isotype control: FPR2‐Alexa647(2.5 μL, sc‐57,141, Santa Cruz), ChemR23‐Alexa488(2.5 μL, sc‐374,570, Santa Cruz), rabbit GPR18 (0.5 μL, NBP2‐24918, Novous Biologicals), and rabbit BLT1 (0.5 μL, 552,836, BD Bioscience). Rabbit antibodies were followed by 2nd anti‐rabbit Alexa488. Samples were incubated in the dark, on ice, for 30 min. The tubes were then treated with 1× 1‐step Fix/Lyse Solution (Invitrogen) by adding water for 5 s, then 10× 1‐step Fix/Lyse Solution (00–5333, eBioscience) and incubated for an additional 15 min at room temperature. Samples were then centrifuged at 500 *g* for 5 min, resuspended with PBS, and analyzed by flow cytometry (BD FACSVerse, Becton, Dickinson, and Company). Analysis was conducted using FlowJo software (FlowJo, LLC).

### 2.5 Monocyte and neutrophil bacterial phagocytosis

Whole blood samples were obtained in heparin. 25 μL of blood samples were incubated with pHrodo S.Aureus (0.005 μg, P35382, Invitrogen) for 1 h at 37°C or on ice. To each tube CD11b/c (2.5 μL, 12–0110‐82, eBioscience) were added. CD11b/c was used to differentiate monocytes from other cell types. Tubes were then incubated in the dark, on ice for 30 min. The tubes were then treated with 1× 1‐step Fix/Lyse Solution (eBioscience) by adding water 5 s, then 10× 1‐step Fix/Lyse Solution and incubated for an additional 15 min at room temperature. Samples were then centrifuged at 500 *g* for 5 min, resuspended with PBS, and analyzed by flow cytometry (BD FACSVerse, Becton, Dickinson, and Company). Analysis was conducted using FlowJo software (FlowJo, LLC).

### 2.6 Reverse transcription polymerase chain reaction (PCR)

We immediately stored specimens in RNAlater after harvest. We isolated total RNA using a RNeasy Micro Kit (Qiagen, Germantown, Md) with RNase‐free DNase treatment according to the manufacturer's protocol. We used total RNA from each specimen to generate complementary DNA using the high –Capacity cDNA Reverse Transcription Kit (Applied Biosynthesis, Foster City, California) for subsequent reverse transcription‐PCR reactions. We detected SYBR Green‐amplified DNA by incorporation of SYBR Green (Applied Biosynthesis). We performed dissociation curve analyses to confirm the specificity of the SYBR Green signal. The PCR products of SPM receptors were confirmed by sequencing and BLAST. Actual primer sequences used for each target gene are shown in Table S1. PCR parameters included an initial 10‐min denaturation step at 95°C, followed by a cycling program of 95°C for 10 s and 60°C for 30 s for 40 cycles (CFX96 Real‐Time System; Bio‐Rad, Hercules, California). We normalized data to a reference gene and subsequently to intact carotid arteries, using delta–delta method.

### 2.7 Immunocytochemistry

We utilized VSMCs and HUVEC grown to 80% confluency in 24 well plates. The cells were activated with a cytokine cocktail including TNFα 10 ng/mL, IL1β 1 μg/mL, and IFNγ 50 ng/mL for 4 h. After incubation in 10% fetal bovine serum (FBS) media for 4 h, we fixed the cells with 4% PFA for 20 min. Cells were permeabilized with 0.2% Triton X‐100 (Sigma‐Aldrich, St. Louis, Missouri) and blocked in UltraCruz Blocking reagent (Santa Cruz Biotechnology, Dallas, Texas). We incubated the cells with primary antibodies: mouse‐anti‐FPR2 (1:50, sc‐57,141, Santa Cruz Biotechnology) or mouse‐anti‐ChemR23 (1:200, sc‐374,570, Santa Cruz Biotechnology) at 4°C overnight. We incubated with a goat‐anti‐mouse Alexa fluor 488 secondary antibody (1:200, A11001, Invitrogen, Waltham, Massachusetts) for 1 h, followed by incubation for 1 h with mouse anti‐actin alpha smooth muscle—Cy3 antibody (1:200, c6198, Sigma Aldrich) for VSMC or with rabbit anti‐CD31 antibody (1:200, orb10314, Biorbyt, St. Louis, Missouri) for HUVEC. We mounted the cells with a 4′6‐diamidino‐2‐phenylindole (DAPI) mounting solution (Southern Biotech, Birmingham, Alabama).

### 2.8 Immunohistochemistry

We processed paraffin‐embedded tissues and took 5 μm sections from the artery. Vessel sections were deparaffinized in xylene, then rehydrated before antigen retrieval with a sodium citrate buffer (Sigma‐Aldrich) using a hot bath to heat to achieve 96°C for 30 min. We permeabilized the sections with 0.2% Triton X‐100 (Sigma‐Aldrich) and blocked in UltraCruz Blocking reagent (Santa Cruz Biotechnology). We incubated the sections with primary antibodies: polyclonal rabbit‐anti‐FPR2 (1:100, NLS1878, Novus Biologicals), polyclonal rabbit‐anti‐ChemR23 (1:100, MBS9701692, MyBioSource.com), polyclonal rabbit‐anti‐CD45 (1:100, ab10558, Abcam), polyclonal rabbit‐anti‐CD31(1:100, NB100‐2284, Novus Biologicals). We incubated the sections with a goat‐anti‐mouse or rabbit Alexa fluor 488 secondary antibody (1:200, A11001, A11008, Invitrogen, Waltham, Massachusetts), followed by incubation for 1 h with mouse anti‐actin alpha smooth muscle—Cy3 antibody (1:200, c6198, Sigma Aldrich). We mounted the sections with a 4′6‐diamidino‐2‐phenylindole (DAPI) mounting solution (Southern Biotech).

### 2.9 Statistical analysis

We performed initial comparisons between groups using a one‐way analysis of variance on all data sets. We used paired two‐tailed Student's *t*‐tests for individual group comparisons of plasma lipid mediator profiling and flow cytometry data of circulating leukocytes, or nonpaired two‐tailed Student's *t*‐tests for individual group comparisons in lipid mediator profiling analysis of the tissues and SPM receptor mRNA expression analysis of cells and tissues, with Bonferroni correction for multiple comparisons.

## 3 RESULTS

### 3.1 Dynamic changes in LM profiles of plasma after vascular injury in rats

**FIGURE 1:**
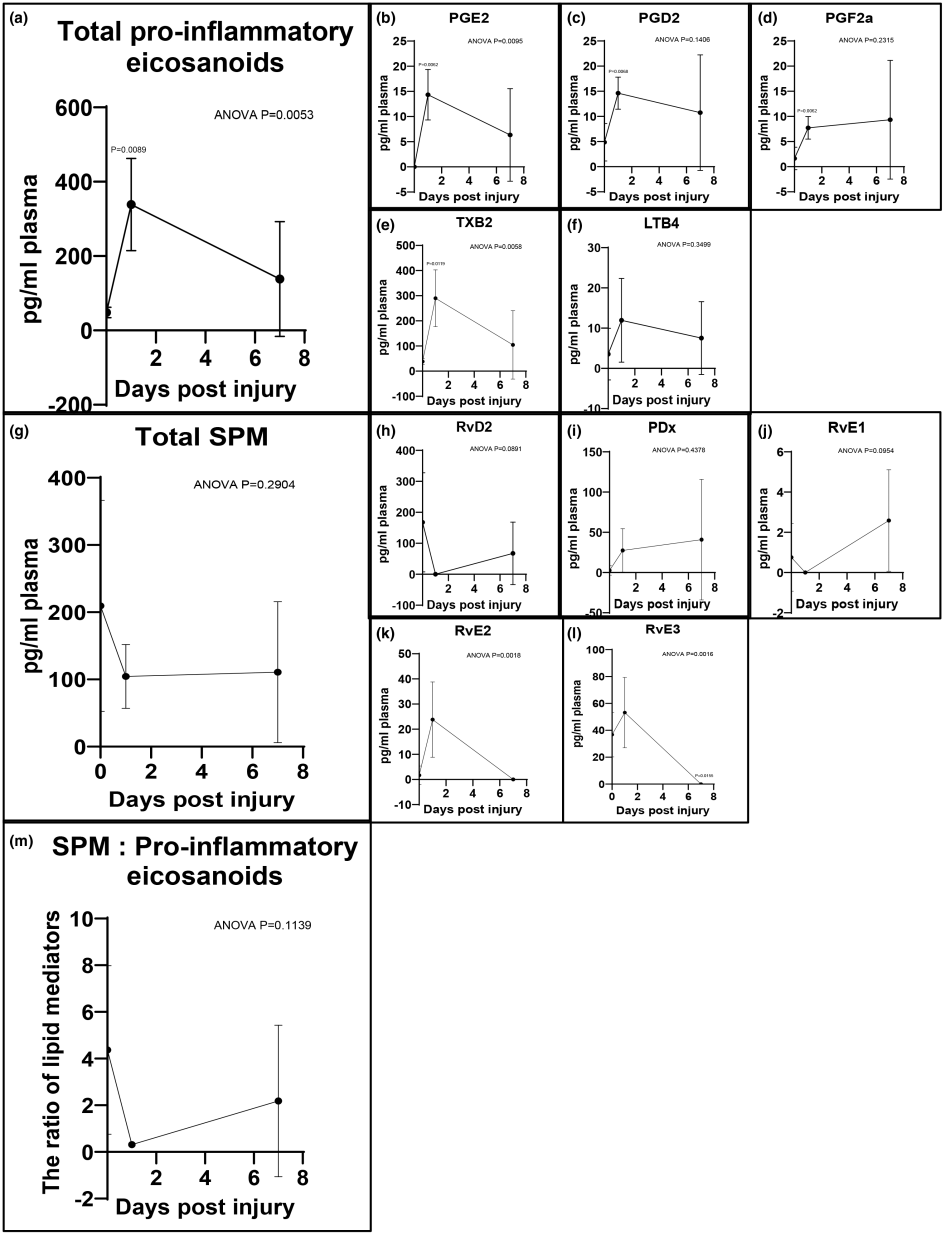
Lipid mediator profiling of plasma after acute vascular injury. Targeted LC–MS/MS analysis of rat plasma collected at baseline, 1 and 7 days after vascular injury. Total pro‐inflammatory eicosanoids include PGE_2_, PGD_2_, PGF_2a_, TXB_2_, and LTB_4_ (a–f). Total SPM includes RvD2, PDx, RvE1, RvE2, and RvE3 (g–l). The ratio of total SPM to total pro‐inflammatory eicosanoids was calculated (m). Results were mean ± SD, *n* = 5 at each time point. *p*‐Values were compared to baseline.

Targeted LC–MS/MS analysis was performed on rat plasma collected at baseline, 1 and 7 days after angioplasty, to evaluate the effect of vascular injury on systemic levels of LMs over time (Figure 1, and Table S2). Levels of pro‐inflammatory eicosanoids in plasma, such as PGE_2_ and TXB_2_, increased sharply at day 1 after injury, then decreased by day 7 though not completely to baseline (Figure 1a,b,e). In contrast, levels of specialized proresolving lipid mediators (SPM) in plasma, including RvD2, PDx, and E‐series resolvins had variable individual patterns but the total plasma SPM level (combination of RvD2, PDx, RvE1, RvE2, and RvE3) was reduced at both day 1 and day 7 compared with baseline (Figure 1g–l). As a result, the ratio of proresolving to pro‐inflammatory LM in plasma nadired at day 1 after vascular injury, then improved marginally by day 7 (Figure 1m **)**.

Levels of intermediate pathway markers of AA pathway in plasma, such as 15‐HETE, also increased at day 1 after injury, then reduced by day 7 though not completely to baseline (Table S2A). In contrast, the levels of intermediate pathway markers of DHA and EPA pathways in plasma, including 17‐HDHA, 18‐HEPE, and 15‐HEPE had variable individual patterns (Table S2B,C). Collectively, these findings document an early systemic inflammatory LM response to arterial injury, and the LM class switch from pro‐inflammatory LM toward SPM occurring in delayed fashion at day 7.

### 3.2 Circulating leukocyte SPM receptor expression and phagocytic activity

**FIGURE 2:**
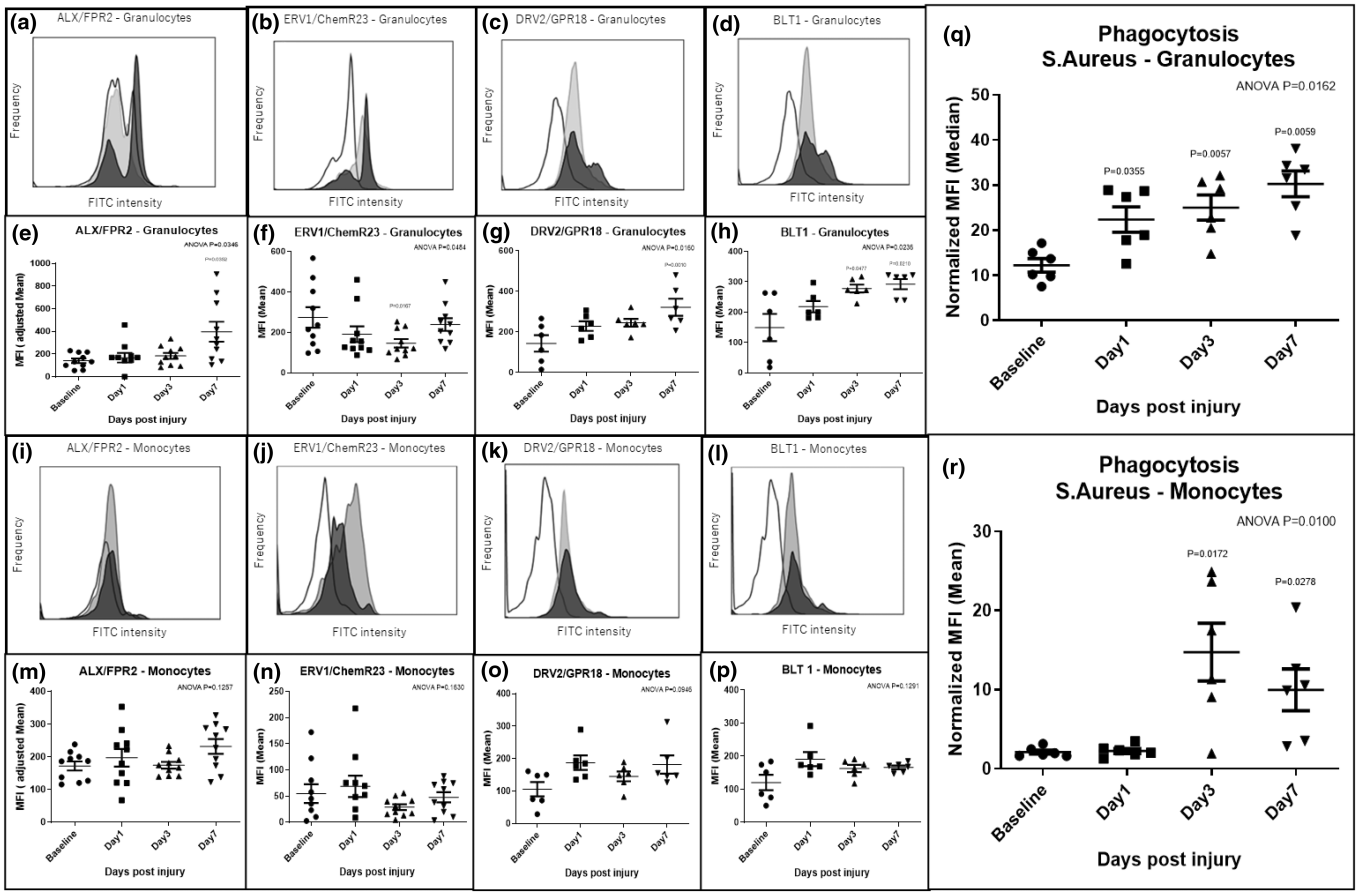
Differential patterns of SPM receptor expression in circulating leukocytes and bacterial phagocytosis activity. SPM receptor expression in rat leukocytes at baseline, 1, 3 and 7 days after vascular injury was assessed by flow cytometry (a–p). Analysis demonstrated higher expression of ALX/FPR2, DRV2/GPR18, and BLT1 protein on granulocytes at day 7 after injury versus baseline (e, g, h), while there was transient depression of ERV1/ChemR23 expression in granulocytes at day 3 after injury (f). Phagocytosis assay (*S. Aureus*) demonstrated early acute increases in activity after vascular injury (q, r). Blank: Baseline, Light gray: Day 1, Dark gray: Day 7 in histogram (a–d, i–l). Results were mean ± SD. ALX/FPR2 and ERV1/ChemR23 (e, f, m, n): *N* = 10 at each time point, DRV2/GPR18, BLT1 (g, h, o, p) and bacterial phagocytosis activity (q, r): *N* = 6 at each time point. *p*‐Values were compared to baseline.

Circulating leukocytes collected at baseline, 1, 3, and 7 days after injury were assessed by flow cytometry to evaluate SPM receptor expression and phagocytic activity, an important physiologic measure of resolution phenotype (Figure 2). Granulocyte expression of ALX/FPR2, DRV2/GPR18, and BLT1 increased postinjury up to day 7 after angioplasty (Figure 2e,g,h), while ERV1/ChemR23 expression was reduced early then recovered by day 7 (Figure 2f). Phagocytic activity of granulocytes to *S. Aureus* increased acutely after injury, beginning 1 day after angioplasty (Figure 2q), while that of monocytes increased in a slightly more delayed fashion, beginning 3 days after angioplasty (Figure 2r).

### 3.3 LM and biosynthetic enzyme profiling of arteries after vascular injury

**FIGURE 3:**
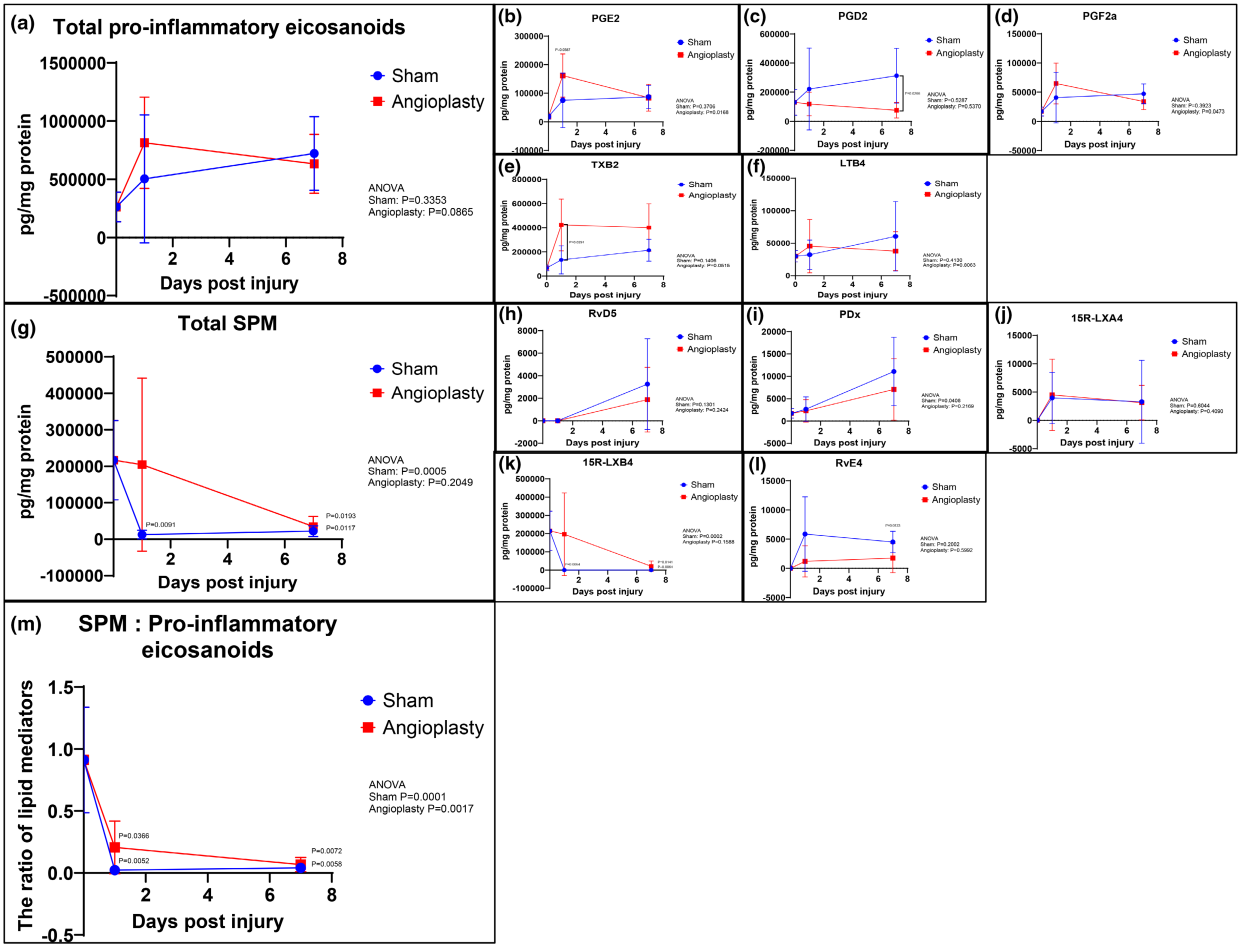
Lipid mediator profiling of arteries after acute vascular injury. Targeted LC–MS/MS of arteries collected at baseline, 1 and 7 days after vascular injury. Total pro‐inflammatory eicosanoids include PGE_2_, PGD_2_, PGF_2a_, TXB_2_, and LTB_4_ (a–f). Total SPM includes RvD5, PDx, 15*R*‐LXA_4_, 15*R*‐LXB_4_, and RvE4 (g–l). The ratio of total SPM to total pro‐inflammatory eicosanoids was calculated (m). Results were mean ± SD. Baseline: *N* = 5, Sham: *N* = 5 at each time point, Angioplasty: *N* = 5 at each time point. *p*‐Values were compared to baseline and between groups at each time point.

Targeted LC–MS/MS analysis of rat arteries collected at baseline, 1 and 7 days after angioplasty was performed to evaluate the temporal profile of LMs in vascular tissue (Figure 3, and Table S3). The analysis demonstrated that levels of pro‐inflammatory eicosanoids (e.g., PGE_2_ and TXB_2_) increased acutely in surgically manipulated arteries (including sham), particularly in the balloon angioplasty group. Eicosanoid levels were sharply increased at day 1 after injury and remained elevated through day 7 (Figure 3a,b,e). In contrast, the level of SPM, including RvD5, PDx, 15*R*‐LXA_4_, and RvE4 were minimal to undetectable 1 day after surgical manipulation in both sham and angioplasty groups, and just trending upwards at 7 days (Figure 3h,i,j). As a result, the ratio of proresolving to pro‐inflammatory LM measured in arteries dropped severely 1 day postinjury and remained markedly depressed through day 7 (Figure 3m **)**.

Levels of intermediate pathway markers of AA pathway in arteries, such as 15‐HETE also increased after injury, and remained elevated through day 7 (Table S3A). In contrast, the levels of intermediate pathway markers of DHA and EPA pathways in arteries, including 17‐HDHA and 15‐HEPE, were minimal 1 day after angioplasty and just trending upward at 7 days (Table S3B,C).

LMs are generated locally by transcellular synthetic pathways involving sequential oxygenation of fatty acid precursors by lipoxygenase (LOX) and cyclo‐oxygenase (COX) enzymes. qRT‐PCR was performed to evaluate the relative mRNA expression of 5‐LOX and COX‐2, two key enzymes involved in LM synthesis, within the vessel wall (Figure S1). Both 5‐LOX and COX‐2 were detected within rat arterial tissues at all time points. Expression levels sharply increased 4 h postinjury. Local 5‐LOX expression increased after angioplasty and remained high up to day 3 versus sham.

### 3.4 SPM receptor expression in normal and injured vascular tissues

**FIGURE 4:**
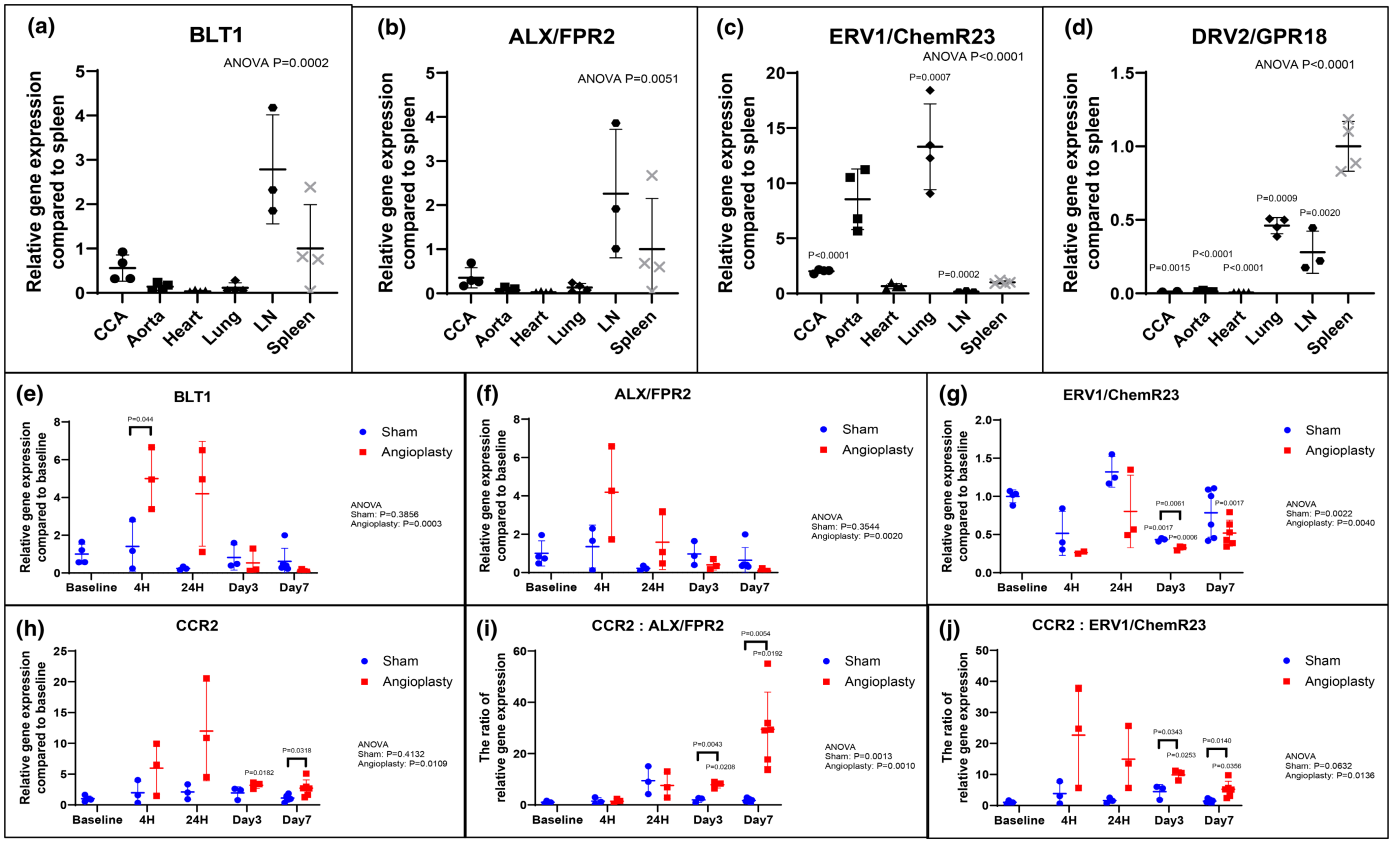
SPM receptor and pro‐inflammatory marker expression in normal and injured vascular tissues. qRT‐PCR demonstrated variable SPM receptor expression levels in analyzed rat organs (a–d). ERV1/ChemR23 mRNA expression of CCA and aorta were 2.0 and 8.5 times higher than that of spleen, respectively (c). In contrast, DRV2/GPR18 mRNA expression of CCA was 1% of that of spleen (d). Results were mean ± SD. CCA, Aorta, Heart, Lung, Spleen: *N* = 4, LN: *N* = 3. *p*‐Values were compared to spleen. qRT‐PCR demonstrated SPM and pro‐inflammatory marker receptor mRNA from rat arteries collected at baseline, 4 h, 1, 3 and 7 days after vascular injury (e–h). Significant dynamic changes were seen especially following balloon angioplasty. BLT1 mRNA expression of the angioplasty group was 3.5 times higher than that of the sham group at 4 h after injury (e). ALX/FPR2 mRNA expression of the angioplasty group was minimum at day 7 after injury, with a transient elevation at 4 h after injury (f). ERV1/ChemR23 mRNA expression of the angioplasty group was 32% and 52% of that of the baseline group at day 3 and day 7 after injury (g). In contrast, CCR2 mRNA expression of the angioplasty group increased sharply after vascular injury, peaked at 24 h, and still remained 2.5 times higher than that of the sham group at day 7 after vascular injury (h). Results were mean ± SD. Baseline: *N* = 4, Sham: *N* = 3 at 4 h, 24 h, Day 3, and *n* = 6 at Day 7, Angioplasty: *N* = 3 at 4 h, 24 h, Day 3, and *n* = 6 at Day 7. *p*‐Values were compared to baseline and between groups at each time point. The ratio of pro‐inflammatory (CCR2) receptor to ALX/FPR2 and ERV1/ChemR23 expression of the angioplasty group were 4.1 and 2.3 times higher at day 3 after injury, and 17.2 and 3.7 times higher at day 7 after injury than that of the sham group, respectively (i, j). Results were mean ± SD. Baseline: *N* = 4, Sham: *N* = 3 at 4 h, 24 h, Day 3, and *n* = 6 at Day 7, Angioplasty: *N* = 3 at 4 h, 24 h, Day 3, and *n* = 6 at Day 7. *p*‐Values were compared to baseline and between groups at each time point. CCA indicated common carotid artery. LN indicated lymph node.

To evaluate the relative tissue distribution of SPM receptors in rats, ALX/FPR2, DRV2/GPR18, and ERV1/ChemR23 mRNA expression in various organs was assessed with qRT‐PCR (Figure 4a–d). We additionally evaluated expression of BLT1, a receptor for LTB_4_ that is also a receptor for E‐series resolvins. All four qPCR products for the receptors were sequenced and their validity confirmed against published rat sequences. Each of the four receptors displayed unique tissue expression patterns in rats. ERV1/ChemR23 expression was highest in lung and vascular tissue, including CCA and aorta (2‐ and 8.5‐fold higher than spleen, respectively; Figure 4c). On the other hand, DRV2/GPR18 mRNA expression in normal cardiovascular tissues, including CCA, aorta and the heart, was minimal (respectively 1, 1, and 0.5% of that detected in the spleen; Figure 4d). Interestingly, the distribution patten of ALX/FPR2 was notably similar to that of BLT1 (Figure 4a,b) with highest levels observed in lymph node and spleen and minimal levels in uninjured arteries.

We evaluated SPM receptor expression in rat carotid arteries at baseline, 4 h, 1, 3 and 7 days after angioplasty (Figure 4e–h). Each SPM receptor showed unique arterial expression patterns in the acute setting. BLT1 mRNA expression increased acutely after angioplasty, remained high at day 1, then returned to baseline levels at day 3 (Figure 4e). ALX/FPR2 mRNA expression acutely increased after injury but returned to baseline between day 1 and day 3, then decreased below baseline through day 7 (Figure 4f). ERV1/ChemR23 mRNA expression in arteries also decreased after injury, but it lacked the early rise seen with ALX/FPR2 and BLT1 (Figure 4g). DRV2/GPR18 mRNA expression in arteries increased after angioplasty, although the measured expression level was still low throughout the course (Figure S2a). In contrast, expression of the pro‐inflammatory CCR2 receptor (a receptor for monocyte chemoattractant protein‐1) increased acutely after angioplasty and remained at markedly elevated levels through day 7, while that of the sham group did not show a clear dynamic change after surgery (Figure 4h). The expression patterns of related pro‐inflammatory cytokines, including IL‐1β and MCP‐1, were similar to that of CCR2: they increased after injury, peaked at day 1, and remained notably elevated 7 days after arterial injury (Figure S2b,c).

To better illustrate relationships between pro‐inflammatory and SPM receptor expression in vascular tissues early after injury, the ratios of CCR2 to ALX/FPR2 and ERV1/ChemR23 mRNA expression were examined at the various time points after angioplasty (Figure 4i,j). As shown in Figure 4i, the ratio of CCR2 to ALX/FPR2 mRNA increased steadily in arterial tissues through day 7 after angioplasty. The ratio of CCR2 to ChemR23 mRNA also increased acutely after angioplasty, then declined gradually but remained elevated above sham on day 7 (Figure 4j). Overall, these data suggest an imbalance of pro‐inflammatory versus proresolving receptor levels in the acute phase after vascular injury, persisting through 7 days.

### 3.5 SPM receptor localization in normal and injured vascular tissues

**FIGURE 5:**
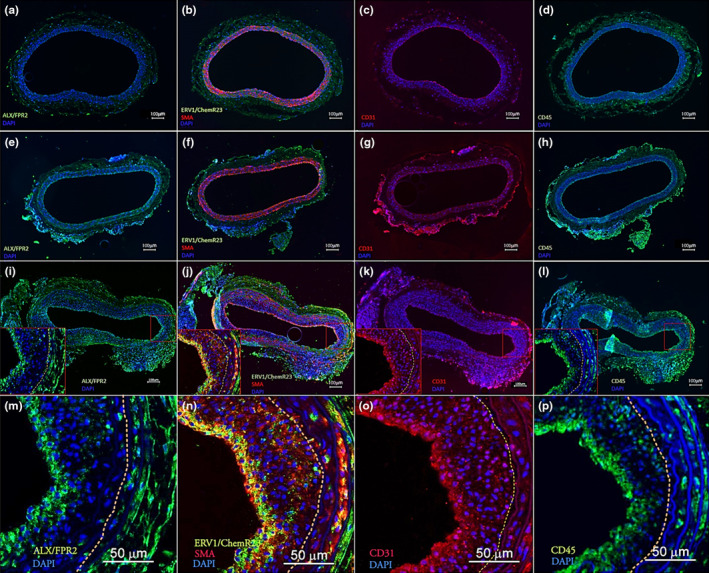
SPM receptor localization in normal and injured vascular tissues. Representative images of immunohistochemical staining of normal and injured arteries were demonstrated. Expression of ALX/FPR2 and ERV1/ChemR23 along with CD31, SMA, and CD45 were assessed at different time points; before (a–d), 1 day (e–h) and 7 days (i–l) after angioplasty. Higher magnification images of injured arteries at 7 days with confocal microscopy demonstrated positive cells of ALX/FPR2, ERV1/ChemR23, CD31 and CD45 in the neo‐endothelial layer (m–p). Dotted line shows the internal Elastic Lamina which defined the border of the neointimal hyperplasia (NIH). Consecutive sections were used for the immunostaining with each antibody in the tissues. SMA indicated smooth muscle Actin. Scale bar: 100 μm (a–l), 50 μm (m–p). Green = ALX/FPR2 or ERV1/ChemR23 or CD45, Red = CD31 or SMA, Blue = DAPI.

Immunostaining of rat arteries with SPM receptor antibodies, including ALX/FPR2 and ERV1/ChemR23, combined with vascular cell and leukocyte markers, identified unique patterns at each time point. At baseline, CD31‐positive cells were lined in the endothelial layer, while few positive cells of ALX/FPR2 or ERV1/ChemR23 were demonstrated largely in the adventitial layer of normal arteries (Figure 5a–c). In injured arteries, complete loss of endothelium was evident on day 1 after angioplasty (Figure 5g). Some positive cells of ALX/FPR2 and CD45 were observed mainly in the adventitia, whereas few positive cells of ERV1/ChemR23 were observed at day 1 (Figure 5e,f,h). CD45‐positive cells were also detected in the media and adventitial layer with confocal imaging (Figure S3a).

Neointimal hyperplasia (NIH) was demonstrated in scattered areas of the injured arteries on day 7 after angioplasty (Figure 5i–l). In these arteries, positive cells of ALX/FPR2, ERV1/ChemR23, CD31, and CD45 were confirmed in the neo‐endothelial and subintimal layer (Figure 5i–p). Positive cells of ALX/FPR2 and ERV1/ChemR23 were also detected in the adventitia, where some positive cells of CD31 might overlap with CD45. With confocal imaging, SPM receptor‐positive cells were detected within the developing neointima at day 7, and scattered positive staining for ALX/FPR2 and ERV1/ChemR23, along with SMA, was detected in the media (Figure 5m–p). Positive staining for ALX/FPR2 in the spleen, and ERV1/ChemR23 and CD31 in the aorta were used as positive controls for these antibodies (Figure S3b–d). Representative images of spleen, aorta, and normal and injured rat arteries immunostained with rabbit IgG were shown as negative control (Figure S3e–i).

### 3.6 SPM receptor expression in cultured vascular cells

**FIGURE 6:**
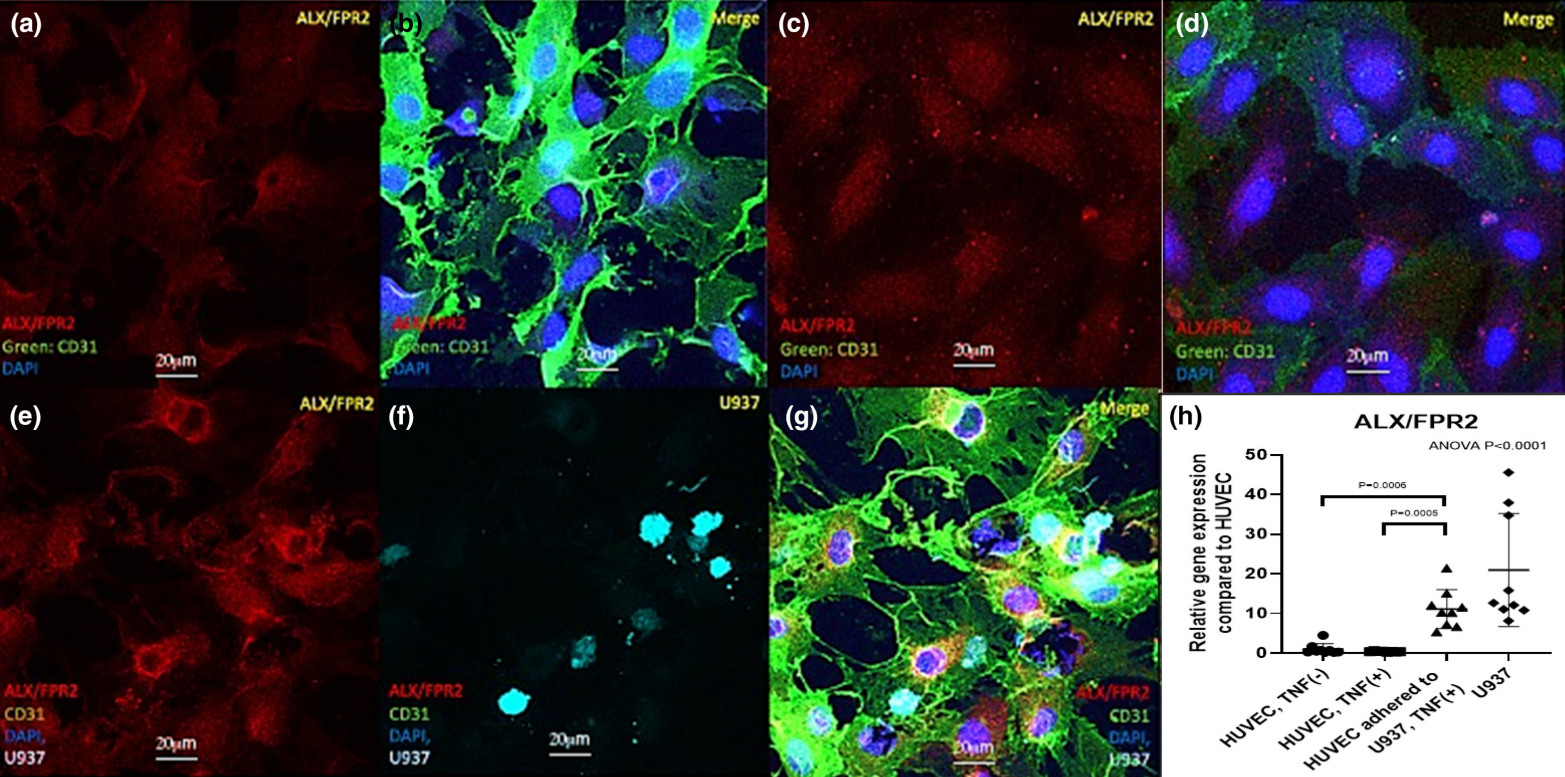
ALX/FPR2 expression in cultured HUVEC. Representative confocal immunofluorescence staining of HUVEC with (c, d) or without (a, b) cytokine stimulation and following U937 adhesion (e–g) were shown, expressing ALX/FPR2. Red = ALX/FPR2, Green = CD31, Blue = DAPI. ALX/FPR2 mRNA expression in HUVEC with or without cytokine stimulation or U937 adhesion was assessed by qRT‐PCR (h). ALX/FPR2 mRNA expression in HUVEC adhered to U‐937 cells was 11.1 times higher than that in HUVEC without U‐937 cells. Results were mean ± SD. HUVEC‐TNF(−), HUVEC adhered to U937‐TNF(+), U937: *N* = 9, HUVEC‐TNF(+): *N* = 8. *p*‐Values were compared between groups. Scale bar: 20 μm. Red = ALX/FPR2, Green = CD31, Blue = DAPI, Cyan = U937.

To evaluate SPM receptor expression in vascular cells in‐vitro, ERV1/ChemR23 and ALX/FPR2 protein and mRNA expression in VSMC and HUVEC were assessed with immunofluorescence staining and qRT‐PCR. Immunostaining and qRT‐PCR (data not shown) confirmed ERV1/ChemR23 protein and mRNA expression in VSMC (Figure S4a,b), which did not change with TNF‐α stimulation (Figure S4c,d). On the other hand, immunostaining displayed limited ALX/FPR2 protein expression in VSMC, both with (Figure S4e,f) and without TNFα stimulation (Figure S4g,h). Minimal expression of both ALX/FPR2 (Figure 6a,b) and ERV1/ChemR23 (data was not shown) was detected in cultured HUVEC without stimulation.

To examine how leukocyte‐EC interactions might influence SPM receptor expression in EC, we utilized an established in‐vitro adhesion model of HUVEC with monocytoid U‐937 cells. In this model, confocal images and qRT‐PCR demonstrated that ALX/FPR2 protein and mRNA expression in HUVECs did not change with cytokine stimulation alone but increased concomitant with U‐937 cell adhesion (Figure 6c–h). Immunocytochemical images suggested that HUVEC in close proximity to adherent U937 demonstrated the strongest signal for ALX/FPR2 expression (Data S1).

## 4 DISCUSSION

This study assessed temporal changes of endogenous bioactive LM, including specialized SPM and their receptors in blood and vascular tissues following acute vascular injury using a rat carotid angioplasty model. Our results demonstrate profound induction of pro‐inflammatory signals in blood and vascular tissues, and for the first time document the induction of SPM circuits and receptors following experimental angioplasty. A deeper understanding of these molecular pathways is needed to develop effective approaches to enhance resolution in the context of acute arterial injury.

Our findings demonstrated dynamic changes in LMs and their receptors in both circulating blood and the arterial wall in the setting of an acute arterial injury. The acute response was characterized by increases in prostaglandins, thromboxanes, and pro‐inflammatory mediators/receptors. A LM class switch toward SPM was evident at 1 week, but the vascular inflammatory response was still profound. One‐week postangioplasty the levels of total SPM in the injured artery remained markedly depressed from baseline, highlighting a potential deficit in resolution signaling. Levy et al. suggested the LM “class switch” theory in their study demonstrating that eicosanoid biosynthesis in human peripheral blood polymorphonuclear neutrophil (PMN) was switched from LTB_4_, a pro‐inflammation lipid mediator, to LXA_4_, proresolution lipid mediator, during acute inflammation (Serhan et al., 2004). Several other studies discovered dynamic changes of proresolution lipid mediators in various models of self‐limited inflammation. For example, a switch from pro‐inflammatory leukotrienes and prostaglandins to SPM is evident in skeletal muscle injury (Giannakis et al., 2019). Moreover, Dalli et al. found temporal distinctions within the family of D‐series resolvins in mice undergoing peritonitis induced by zymosan injection (Dalli, Winkler, et al., 2013). Our study clarified that eicosanoids derived from arachidonic acid (AA) increased acutely in plasma and arteries after angioplasty, reflecting a profound early inflammatory response locally and systemically. In this rat model we found that SPM derived from both docosahexaenoic acid (DHA) and eicosapentaenoic acid (EPA) increased notably in a delayed fashion, especially in arteries, suggesting the LM “class switch” has just begun to take place.

Several SPM receptors have now been identified (Chiang & Serhan, 2020) but their regulation and function remain areas of investigation. We focused on four known SPM receptors for lipoxins, D‐ and E‐series resolvins which have been shown to have direct relevance to vascular injury across a range of in‐vivo models. More recently other SPM receptors (e.g., for PD1) have been defined (Park et al., 2020). BLT1 is known to be a receptor for LTB_4_, working as an agonist, and RvE1 and RvE2, working as antagonists (Serhan & Chiang, 2013). ALX/FPR2 is a receptor for LXA_4_, 15R‐LXA_4_, and RvD1, working as proresolution agonists (Serhan & Chiang, 2013). ERV1/ChemR23 is a receptor for RvE1 and RvE2, working as proresolution agonists (Serhan & Chiang, 2013). Spatial and temporal distribution of SPM receptor expression are of great interest in health and disease, and their contribution to the resolution of vascular inflammation has been an area of focus (Fredman & Spite, 2017; Pirault & Back, 2018).

In these in‐vivo studies, we observed notable differences in the temporal response between the circulating leukocytes and the local arterial wall. Leukocyte phenotype changes occurred early and showed evidence of increased SPM receptor expression and phagocytic activity within days of vascular injury. Several studies have demonstrated SPM receptors expressed on leukocytes. Fluorescence activated cell sorting (FACS) analysis showed ALX/FPR2 expression on neutrophils (Norling et al., 2012) and monocytes (Waechter et al., 2012). Herova et al. indicated ERV1/ChemR23 expression on macrophages (Herova et al., 2015). Chiang et al. demonstrated that DRV2/GPR18 is expressed both on PMN and monocytes (Chiang et al., 2015). However, few studies evaluated SPM receptor expression and their dynamic changes on circulating leukocytes after surgical intervention. We observed that several SPM receptors, such as BLT1, ALX/FPR2, and DRV2/GPR18 expression on circulating granulocytes increased after vascular injury. The dynamic change showed a clear increasing trend up to day 7 after injury, implying systemic reaction to vascular injury. Based on the results of SPM biosynthesis after injury, we hypothesized that a systemic resolution response would precede the local response. Further work is needed in disease models and human studies, particularly in the context of atherosclerosis, to better clarify these dynamic and interrelated changes in blood and tissue.

Expression of SPM receptors in the arterial wall occurred in a more delayed fashion than on circulating leukocytes. In arterial tissue, the temporal expression patterns of each of the examined SPM receptors were unique. BLT1 expression in injured arteries increased acutely after angioplasty, indicating a local acute inflammatory response. Interestingly, ALX/FPR2 expression in injured arteries demonstrated a transient increase after angioplasty but decreased soon after the peak at 4 h. On the other hand, ERV1/ChemR23 expression in injured arteries decreased without the transient increase after angioplasty. These discrepant SPM receptor expression patterns are notable in relation to the different characteristics of pathways: BLT1 is targeted by inflammatory mediators such as LTB_4_, whereas ALX/FPR2 and ERV1/ChemR23 are activated by proresolution mediators such as D‐and E‐ series resolvins. A few studies displayed time‐course analysis of SPM receptors in tissue injury. Sansbury et al. demonstrated a dynamic change of RvD1 and ALX/FPR2 in the bone marrow and skeletal muscle in mice with hind limb ischemia (Sansbury & Spite, 2016). Kain et al. found that ALX/FPR2 mRNA increased in the left ventrical and spleen following experimental myocardial infarction (Kain et al., 2015). However, few studies examined the time‐course analysis of SPM receptors after the surgical intervention simultaneously compared several SPM receptors. The findings of our study indicated that SPM receptor expression changed dynamically and uniquely in the systemic and local responses to arterial injury.

SPM receptor expression in vascular tissues was also evaluated with immunohistochemistry. On day 7 after angioplasty, as the neointima was developing, SPM receptor expression is most notable in the subintimal and adventitial layers, in areas also populated by scattered CD45+ leukocytes. Several studies showed SPM receptor expression in vasculature. Ho et al. demonstrated ERV1/ChemR23 expression on vascular smooth muscle cell (VSMC) derived from the human saphenous vein (Ho et al., 2010). Laguna‐Fernandez et al. indicated that ERV1/ChemR23 was expressed in the atherosclerotic carotid artery and colocalized with VSMC (Laguna‐Fernandez et al., 2018). Several other immunohistochemistry studies for human and rodent specimens supported these findings (Jannaway et al., 2018; Kostopoulos et al., 2014; Watts et al., 2013). Similar to the previous reports, our findings on day 7 after angioplasty exhibited ERV1/ChemR23 positive cells in the subintimal layer, co‐localized with smooth muscle actin (SMA), which implied ERV1/ChemR23 expressed on VSMC.

ALX/FPR2 expression in endothelial cells (EC) has been previously suggested in a few studies. Petri et al. demonstrated ALX/FPR2 expressed in the atherosclerotic carotid artery and co‐localized with EC (Petri et al., 2015). They also showed ALX/FPR2 was expressed in the aorta and co‐localized with PMN (Petri et al., 2018). Our results were consistent with these findings. On day 1 after angioplasty, several ALX/FPR2 positive cells were confirmed in the adventitia, co‐localized with CD45. Subsequently, ALX/FPR2 was detected on the EC layer on day 7 after angioplasty, which suggested ALX/FPR2 expression within the re‐endothelialization process that accompanies neointimal hyperplasia (NIH). To validate these findings, we assessed SPM receptor expression on cultured vascular cells. Our in vitro findings suggested that direct leukocyte‐EC interactions might trigger increased local endothelial expression of SPM receptors such as ALX/FPR2, which could provide an intrinsic brake to control the inflammatory process in the vasculature. As Petri et al. demonstrated, clarifying the role of SPM receptors such as ALX/FPR2 and their pathway might benefit pharmacologic therapeutic approaches for vascular diseases such as atherosclerosis and aortic aneurysm (Petri et al., 2015, 2018). Further mechanistic studies are needed to confirm and extend these interesting findings.

Our results demonstrate that an imbalance between pro‐inflammatory and proresolving pathway markers in the angioplasty setting increased and remained high up to day 7 after vascular injury. Recent investigators suggested that atherosclerosis resulted from an imbalance of pro‐inflammatory and proresolution lipid mediators (Back et al., 2019; Kasikara et al., 2018). According to these results, the persistent imbalance between inflammation and resolution in the injured arteries after angioplasty might cause late procedure‐related pathologies, including NIH. Indeed, we have found that local administration of SPM could attenuate NIH created by vascular injury (Akagi et al., 2015; Kim et al., 2022; Miyahara et al., 2013; Wu et al., 2018; Wu, Mottola, Chatterjee, et al., 2017). Clarifying dynamic changes of SPM receptor expression and the balance between pro‐inflammatory and SPM could facilitate the healing of vascular inflammation. Our findings that ERV1/ChemR23 is expressed on neointimal VSMC may be relevant for controlling NIH, which is a culprit for restenosis following vascular interventions (Conte et al., 2006; de Vries et al., 2016; Satish & Agrawal, 2019). However, local availability of SPM receptors, as well as the balance between synthetic and degradation pathways for LMs, are likely to be of great importance and are not well understood.

There are several notable limitations in this study. First, we intentionally focused our study to the acute injury response through 7 days after injury. Although this period was limited, we demonstrate important changes in LM‐pathways in both the systemic and local responses to injury in the acute phase. Further studies that extend the follow‐up period after angioplasty would be relevant to characterize the complete remodeling response. Second, we utilized the male rat angioplasty model which takes place in the setting of a normal healthy artery. In the clinical setting, vascular procedures are utilized in diseased arteries, most commonly from atherosclerosis. Therefore, further study using an injury model created from diseased vessels such as hyperlipidemia or diabetes is warranted. The present studies were conducted to uncover the molecular pathways and mechanisms underlying observations we have previously made on the therapeutic effects of SPM using the identical model of rat carotid angioplasty. In these prior publications (Kim et al., 2022; Levy et al., 2023; Mottola et al., 2020; Wu, Mottola, Chatterjee, et al., 2017), adult male SD rats were used exclusively for these experiments. Further studies are needed to determine if there may be differences in these pathways and responses between males and females. Third, we used the monocytic U937 cell line for the vascular cell adhesion model, based on prior work. Further studies should incorporate freshly harvested circulating monocytes. Although it is technically challenging to make a similar model with PMN, it would be interesting as neutrophils play an essential role in vascular inflammation, especially in the acute phase.

Our data demonstrate acute, time‐dependent changes of lipid mediator biosynthesis and SPM receptor expression in plasma, leukocytes, and artery walls following vascular injury. A persistent imbalance between inflammation and resolution pathways was evident at 7 days after angioplasty in this model. Further studies are needed to elucidate the specific role of SPMs and their receptors in the resolution and healing phase after vascular injury. These investigations will provide the basis for intelligent design of proresolving therapeutics to accelerate vascular healing and improve the outcomes of vascular interventions for patients with advanced peripheral and coronary atherosclerosis.

## AUTHOR CONTRIBUTIONS

Conceived and designed research: Hideo Kagaya, Michael S Conte. Performed experiments: Hideo Kagaya, Mian Chen, Xuanshi Yin, Matthew Spite. Analyzed data: Hideo Kagaya, Alexander S Kim, Mian Chen, Pei‐Yu Lin, Xuanchi Yin, Matthew Spite. Interpreted results of experiments: Hideo Kagaya, Alexander S Kim, Mian Chen, Matthew Spite, Michael S Conte. Prepared figures: Hideo Kagaya, Mian Chen. Drafted manuscript: Hideo Kagaya, Mian Chen, Matthew Spite. Edited and revised manuscript: Hideo Kagaya, Alexander S Kim, Matthew Spite, Michael S Conte. Approved final version of manuscript: Hideo Kagaya, Michael S Conte.

## FUNDING INFORMATION

Michael S. Conte: HL, 119508; Matthew Spite: HL, 106173.

## ETHICS STATEMENT

This study was approved by the Institutional Animal Care and Use Committee of the University of California, San Francisco which insures that the use of animals in research is both humane and meets ethical standards of scientific conduct.

## DISCLOSURES

Dr. Conte is a co‐inventor on a patent assigned to the University of California San Francisco and Brigham and Women's Hospital. He is also a cofounder of VasaRx.

## Supporting information


Figure S1.

Table S1.

Data S1.

## Data Availability

The data that support the findings of this study are available from the corresponding author, and co‐authors: HK, MCS, and MC upon reasonable request.
