# Supplementary material for: Dynamic changes in proresolving lipid mediators and their receptors following acute vascular injury in male rats
Source: Physiol Rep. 2024 Aug 11;12(15):e16178. doi: 10.14814/phy2.16178 (PMC11317191; doi:10.14814/phy2.16178)
Supplement: Supplementary file 1 — Figure S1. [file PHY2-12-e16178-s003.docx]

**Supplemental Figure 1. Biosynthetic enzyme expression in normal and injured vascular tissues.**

**Supplemental Figure 2. SPM receptor and pro-inflammatory marker expression in normal and injured vascular tissues.**


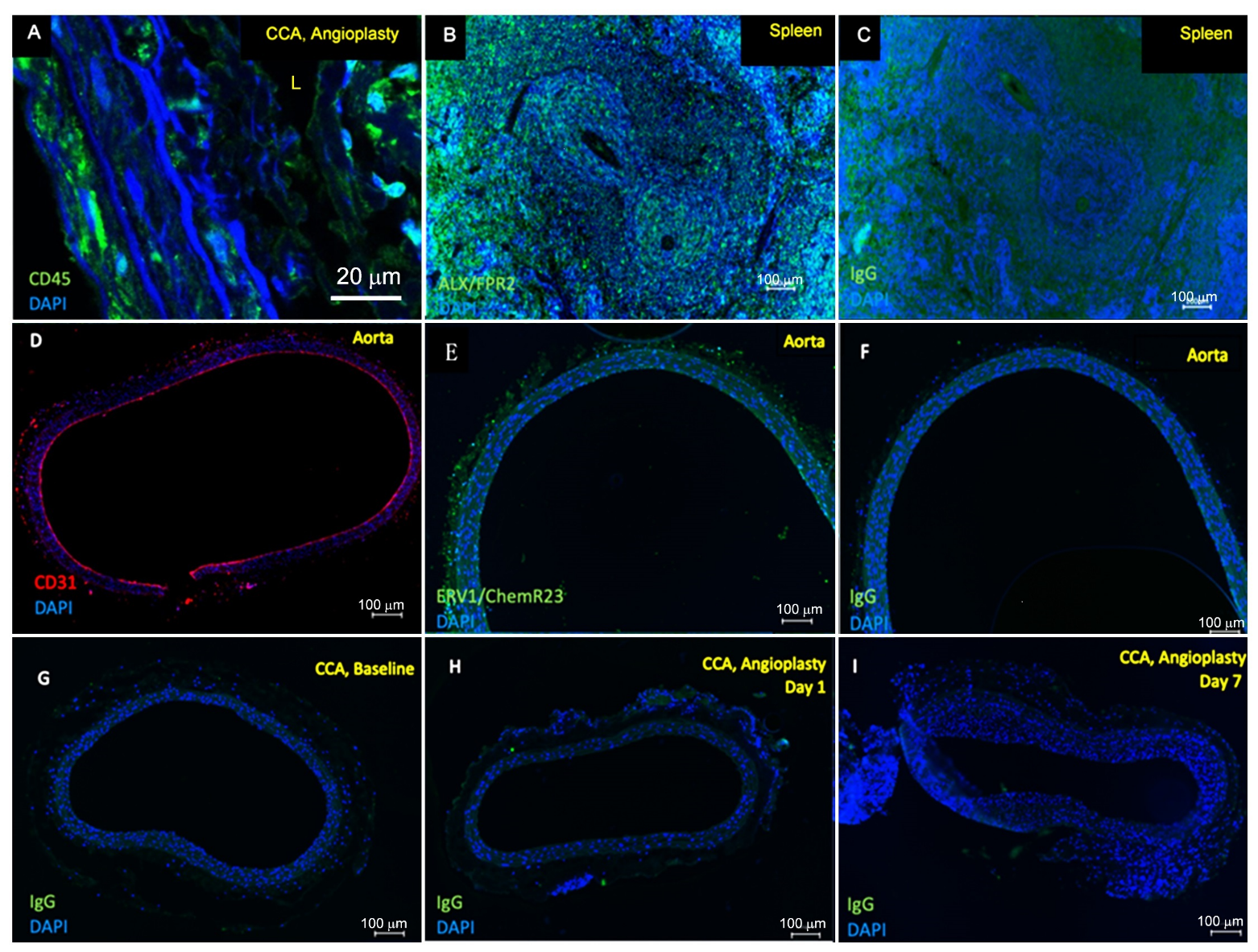


**Supplemental Figure 3. SPM receptor localization in spleen, aorta, and normal and injured vascular tissues.**


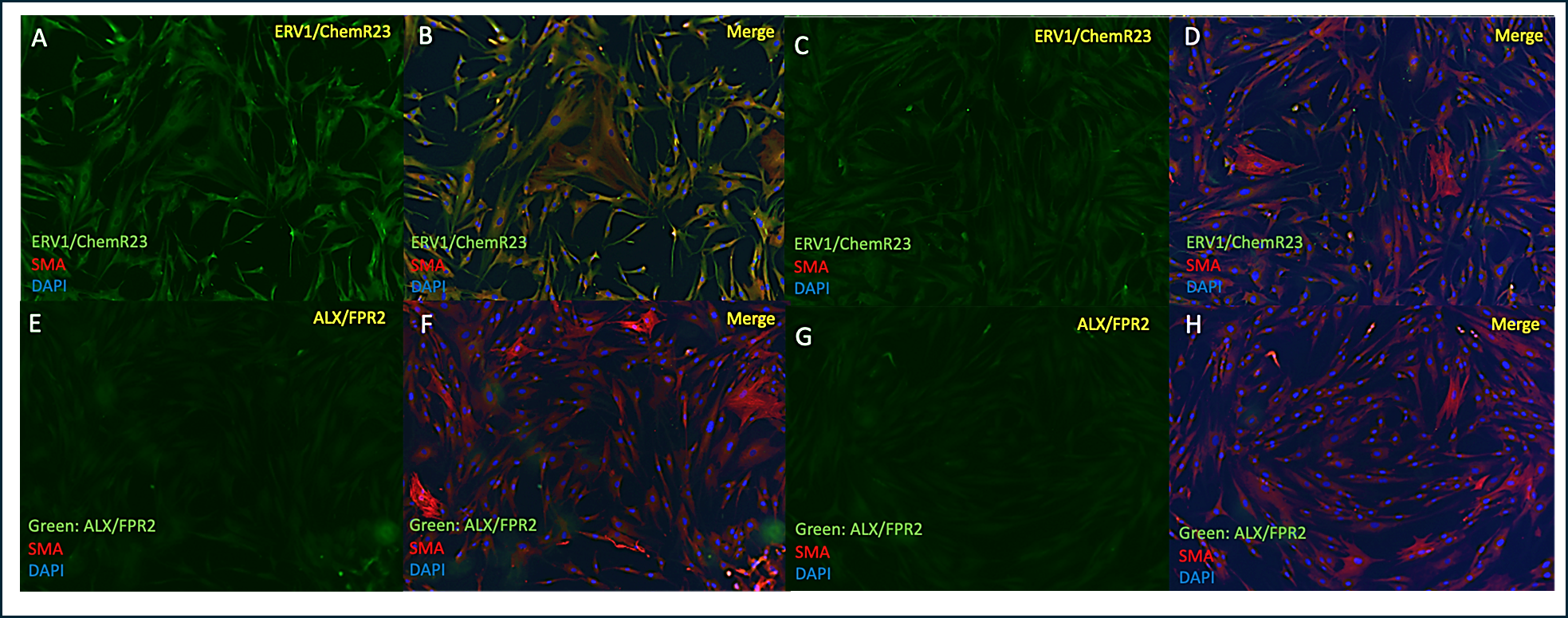


**Supplemental Figure 4. SPM receptor expression in cultured VSMC.**
