# Supplementary material for: Dynamic changes in proresolving lipid mediators and their receptors following acute vascular injury in male rats
Source: Physiol Rep. 2024 Aug 11;12(15):e16178. doi: 10.14814/phy2.16178 (PMC11317191; doi:10.14814/phy2.16178)
Supplement: Supplementary file 2 — Table S1. [file PHY2-12-e16178-s002.docx]

**SUPPLEMENTAL TABLES**

**Supplemental Table 1.** Actual primer sequences used for each target gene.

| Primer | Forward sequence | Reverse sequence |
| --- | --- | --- |
| BLT1 | AGGTATCTGGGTGGTGTCTT | CGTCTTGTTCTTGGGTGTTACT |
| ALX/FPR2 | GTAACAGTTAGAGGGAGCATCAG | CTTGACAGCGATGAGTCCATAG |
| ERV1/ChemR23 | CCAAGTCCACGATGTAGTCC | CGAGTTCTATTCTGCAACAGTG |
| DRV2/GPR18 | ACACTCTTTCCAGCACCTC | TGTATTCCTCTGGGTGTGAAC |
| CCR2 | TGT GAG GCT CAT CTT TGC C | ACC ACA CAG TTA CTC ATT CCC |
| HPRT | TGACCTGCTGGATTACATTAAAGC | GTCATTACAAGTAGCTCTTCAGTCTGATAAA |

**Supplemental Table 2A.** Lipid mediator (LM) profiling of plasma after vascular injury in rats.

Arachidonic acid (AA) derived products.

| AA derived | Baseline (Mean$\boldsymbol{\pm}$SE, pg/ml) | Day 1 (Mean$\boldsymbol{\pm}$SE, pg/ml) | Day 7 (Mean$\boldsymbol{\pm}$SE, pg/ml) |
| --- | --- | --- | --- |
| PGE2 | ND | 14.3$\pm$2.2 | 6.3$\pm$4.1 |
| PGD2 | 4.9$\pm1.7$ | 14.6$\pm1.5$ | 10.7$\pm5.1$ |
| PGF2a | 1.6$\pm1.0$ | 7.7$\pm1.0$ | 9.3$\pm5.3$ |
| TXB2 | 38.0$\pm$4.9 | 289.8$\pm$50.5 | 104.3$\pm$60.8 |
| LXA4 | ND | ND | ND |
| 15R-LXA4 | ND | ND | ND |
| LXB4 | ND | ND | ND |
| 15R-LXB4 | ND | ND | ND |
| LTB4 | 3.5$\pm$2.9 | 11.9$\pm$4.6 | 7.5$\pm$4.0 |
| 15-HETE | 78.2$\pm$19.2 | 242.0$\pm$34.5 | 106.3$\pm$51.9 |
| 12-HETE | 1075.2$\pm228$.5 | 15295.7$\pm4995$.1 | 3311.6$\pm202$1.8 |
| 5-HETE | 181.4$\pm$13.7 | 266.0$\pm28$.2 | 140.7$\pm27$.8 |

**Supplemental Table 2B. Lipid mediator (LM) profiling of plasma after vascular injury in rats.** Docosahexaenoic acid (DHA) derived products.

| DHA derived | Baseline (Mean$\boldsymbol{\pm}$SE, pg/ml) | Day 1 (Mean$\boldsymbol{\pm}$SE, pg/ml) | Day 7 (Mean$\boldsymbol{\pm}$SE, pg/ml) |
| --- | --- | --- | --- |
| RvD1 | ND | ND | ND |
| 17R-RvD1 | ND | ND | ND |
| RvD2 | 167.6$\pm$71.6 | ND | 67.3$\pm$45.2 |
| RvD3 | ND | ND | ND |
| 17R-RvD3 | ND | ND | ND |
| RvD4 | ND | ND | ND |
| RvD5 | ND | ND | ND |
| RvD6 | ND | ND | ND |
| Maresin 1 | ND | ND | ND |
| 12-trans Mar1 | ND | ND | ND |
| 7-epi, 12-trans Mar1 | ND | ND | ND |
| 7S, 14S-diHDHA | ND | ND | ND |
| Maresin 2 | ND | ND | ND |
| PD 1 | ND | ND | ND |
| 17R-PD 1 | ND | ND | ND |
| PDx | 2.6$\pm$2.6 | 27.4$\pm$12.1 | 40.9$\pm$33.5 |
| 17-HDHA | 102.8$\pm$40.1 | 460.0.$\pm$129.3 | 362.0$\pm$282.1 |
| 14-HDHA | 253.8$\pm$42.3 | 2840.$4\pm806$.4 | 1204.9$\pm854$.3 |

**Supplemental Table 2C. Lipid mediator (LM) profiling of plasma after vascular injury in rats.** Eicosapentaenoic acid (EPA) derived products.

| EPA derived | Baseline (Mean$\boldsymbol{\pm}$SE, pg/ml) | Day 1 (Mean$\boldsymbol{\pm}$SE, pg/ml) | Day 7 (Mean$\boldsymbol{\pm}$SE, pg/ml) |
| --- | --- | --- | --- |
| LXA5 | ND | ND | ND |
| RvE1 | 0.8$\pm0.8$ | ND | 2.6$\pm$1.1 |
| RvE2 | 1.7$\pm$1.7 | 23.8$\pm$6.7 | ND |
| RvE3 | 36.8$\pm$7.4 | 53.2$\pm$11.7 | ND |
| RvE4 | ND | ND | ND |
| 18-HEPE | 242.9$\pm$43.1 | 144.6$\pm$20.2 | 150.9$\pm$17.2 |
| 15-HEPE | 131.2$\pm$22.4 | 197.4$\pm$54.3 | 193.9$\pm$108.6 |
| 12-HEPE | 523.0$\pm100$.8 | 7006.1$\pm$2707.1 | 1335.4$\pm69$7.0 |
| 5-HEPE | 231.1$\pm35$.2 | 244.8$\pm$23.3 | 164.1$\pm44$.7 |

**Supplemental Table 3A. LM profiling of arteries after vascular injury in rats.**

Arachidonic acid (AA) derived products.

| AA derived | Baseline (Mean$\boldsymbol{\pm}$SE, pg/mg protein) | Day 1 (Mean$\boldsymbol{\pm}$SE, pg/mg protein)  Sham Angioplasty | | Day 7 (Mean$\boldsymbol{\pm}$SE, pg/mg protein)  Sham Angioplasty | |
| --- | --- | --- | --- | --- | --- |
| PGE2 | 18494.3$\pm$5049.1 | 75305.2$\pm$42508.1 | 161778.2$\pm$33788.8 | 87126.8$\pm$18218.2 | 84305.2$\pm$21018.8 |
| PGD2 | 129806.7$\pm$51131.9 | 221317.4$\pm125682.6$ | 118473.6$\pm$36071.3 | 312638.6$\pm$84018.8 | 75780.2$\pm$23782.9 |
| PGF2a | 16728.1$\pm$4372.4 | 40580.6$\pm$19352.3 | 64843.7$\pm$15669.5 | 47075.8$\pm$7561.5 | 33927.5$\pm$6100.5 |
| TXB2 | 67223.4$\pm$13239.8 | 133775.5$\pm$51890.8 | 422678.0$\pm$95672.6 | 213814.6$\pm$40610.6 | 400637.8$\pm$88195.5 |
| LXA4 | ND | ND | ND | ND | ND |
| 15R-LXA4 | 0 | 0 | 4502.6$\pm$2813.2 | 3280.2$\pm$3280.2 | 3128.9$\pm$1368.1 |
| LXB4 | ND | ND | ND | ND | ND |
| 15R-LXB4 | 215025.0$\pm$62056.8 | 0 | 196402.0$\pm$101333.0 | 0 | 20909.6$\pm$12836.7 |
| LXA5 | ND | ND | ND | ND | ND |
| LTB4 | 29974.2$\pm$.5089.8 | 32251.8$\pm$11943.0 | 45475.4$\pm$9056.9 | 60609.9$\pm$26441.6 | 37811.3$\pm$16553.6 |
| 15-HETE | 277669.7$\pm$4022.2 | 1014452.6$\pm$353355.8 | 422497.8$\pm$101142.7 | 858358.0$\pm$130796.3 | 514132.8$\pm$106033.5 |
| 12-HETE | 554255.0$\pm$93709.9 | 1126395.0$\pm$390868.3 | 1228274.2$\pm$278485.1 | 1828823.6$\pm$279842.1 | 1617092.8$\pm$198684.8 |
| 5-HETE | 18042.4$\pm$3025.6 | 27034.4$\pm$7963.7 | 34170.9$\pm$11074.6 | 31343.1$\pm$4968.7 | 29678.4$\pm$2957.5 |

**Supplemental Table 3B. LM profiling of arteries after vascular injury in rats.**

Docosahexaenoic acid (DHA) derived products.

| DHA derived | Baseline (Mean$\boldsymbol{\pm}$SE, pg/mg protein) | Day 1 (Mean$\boldsymbol{\pm}$SE, pg/mg protein)  Sham Angioplasty | | Day 7 (Mean$\boldsymbol{\pm}$SE, pg/mg protein)  Sham Angioplasty | |
| --- | --- | --- | --- | --- | --- |
| RvD1 | ND | ND | ND | ND | ND |
| 17R-RvD1 | ND | ND | ND | ND | ND |
| RvD2 | ND | ND | ND | ND | ND |
| RvD3 | ND | ND | ND | ND | ND |
| 17R-RvD3 | ND | ND | ND | ND | ND |
| RvD4 | ND | ND | ND | ND | ND |
| RvD5 | 0 | 0 | 0 | 3255.2$\pm$1799.2 | 1873.9$\pm$1284.1 |
| RvD6 | ND | ND | ND | ND | ND |
| Maresin 1 | ND | ND | ND | ND | ND |
| 12-trans Mar1 | ND | ND | ND | ND | ND |
| 7-epi, 12-trans Mar 1 | ND | ND | ND | ND | ND |
| 7S, 14S-diHDHA | ND | ND | ND | ND | ND |
| Maresin 2 | ND | ND | ND | ND | ND |
| PD1 | ND | ND | ND | ND | ND |
| 17R-PD1 | ND | ND | ND | ND | ND |
| PDx | 1710.8$\pm$615.2 | 2659.0$\pm$1223.9 | 2285.6$\pm$1115.4 | 11074.1$\pm$3419.3 | 7072.1$\pm$3081.3 |
| 17-HDHA | 215151.0$\pm$41199.7 | 478926.7$\pm$161268.0 | 269701.1$\pm$128923.4 | 2985713.0$\pm$1984862.7 | 611690.8$\pm$211160.5 |
| 14-HDHA | 201139.0$\pm$57580.6 | 342933.0$\pm$145926.8 | 361844.5$\pm$136645.5 | 957880.0$\pm$180128.7 | 700181.8$\pm$215580.7 |

**Supplemental Table 3C. LM profiling of arteries after vascular injury in rats.**

Eicosapentaenoic acid (EPA) derived products.

| EPA derived | Baseline (Mean$\boldsymbol{\pm}$SE, pg/mg protein) | Day 1 (Mean$\boldsymbol{\pm}$SE, pg/mg protein)  Sham Angioplasty | | Day 7 (Mean$\boldsymbol{\pm}$SE, pg/mg protein)  Sham Angioplasty | |
| --- | --- | --- | --- | --- | --- |
| LXA5 | ND | ND | ND | ND | ND |
| LXB5 | ND | ND | ND | ND | ND |
| RvE1 | ND | ND | ND | ND | ND |
| RvE2 | ND | ND | ND | ND | ND |
| RvE3 | ND | ND | ND | ND | ND |
| RvE4 | 0 | 5963.9$\pm$2848.1 | 1195.5$\pm$1195.5 | 4508.0$\pm$819.2 | 1753.5$\pm$1121.3 |
| 18-HEPE | 1407.2$\pm$36.2 | 5250.0$\pm$1765.3 | 4139.0$\pm$1217.8 | 3150.0.0$\pm$902.0 | 4221.0$\pm$934.4 |
| 15-HEPE | 14476.5$\pm$3654.5 | 44518.9$\pm$15252.9 | 29593.4$\pm$15260.1 | 103965.0$\pm$27212.1 | 55706.9$\pm$20863.7 |
| 12-HEPE | 38119.3$\pm$8545.6 | 82860.8$\pm$28783.6 | 230043.2$\pm$63165.9 | 197867.4$\pm$39728.0 | 252904.2$\pm$30422.0 |
| 5-HEPE | 1404.5$\pm$268.8 | 3089.2$\pm$1597.4 | 6156.2$\pm$2570.6 | 2096.9$\pm$496.8 | 2461.7$\pm$298.5 |
