# Supplementary material for: Dynamic changes in proresolving lipid mediators and their receptors following acute vascular injury in male rats
Source: Physiol Rep. 2024 Aug 11;12(15):e16178. doi: 10.14814/phy2.16178 (PMC11317191; doi:10.14814/phy2.16178)
Supplement: Supplementary file 3 — Data S1. [file PHY2-12-e16178-s001.docx]

Prof. Josephine Adams

Editor-in-chief

Physiological Reports

Prof. Daphne Merkus

Associate Editor

Physiological Reports

RE: PHYSREP 2024-02-086

July 4, 2024

Dear Prof. Josephine Adams and Prof. Daphne Merkus,

I am pleased to resubmit an original article entitled “Dynamic changes in pro-resolving lipid mediators and their receptors following acute vascular injury in male rats” for publication in Physiological Reports. Prior investigations from our group and others have highlighted the role of bioactive lipid mediators (LM), including members of the specialized pro-resolving mediator (SPM) families, in vascular disease. This manuscript builds upon this body or work to characterize the acute phase response of SPM and their receptors in both circulating blood and within the arterial wall following vascular injury.

We thank the reviewers for their insightful comments and questions. In response, we have made significant changes to the figures to better represent the experimental data, as well as multiple changes within the text. We appreciate your patience and believe that these changes have greatly improved the manuscript.

We believe that this manuscript is appropriate for publication by Physiological Reports because it seeks basic and translational investigations that address across all areas of physiology and allied disciplines. Our findings will interest the readers of Physiological Reports and will likely impact the work of others exploring the mechanistic and translational relevance of resolution of inflammation in cardiovascular disease. This manuscript has not been published and is not under consideration for publication elsewhere. We have a few conflicts of interest to disclose:

**List COI to disclose:**

**Dr. Conte is a co-inventor on a patent assigned to the University of California San Francisco and Brigham and Women’s Hospital. He is also a co-founder of VasaRx.**

Thank you again for your consideration of our work. We look forward to your reply.

Sincerely,

Hideo Kagaya, MD, PhD

Cardiovascular Research Institute and Department of Surgery, University of California San Francisco, California, USA
